# Complex chest wall reconstruction after massive leiomyosarcoma resection: a case report on a combined thoracic and plastic surgical team approach

**DOI:** 10.1097/MS9.0000000000005651

**Published:** 2026-09-24

**Authors:** Waseem Sajjad, Fiza Riaz, Asma Ali Akbar, Minal Zaman Cheema, Mohamed Kamal Said, Aseef Rehman, Krithika Nathan, Natasha Khawaja Khail, Ibrahim Salau, Jyoti Kella, Neesha, Ihsan Qamar, Hassan Fahs

**Affiliations:** a Department of Medicine, King Edward Medical University, Mayo Hospital, Lahore, Pakistan; b Department of Medicine, CMH Kharian Medical College, Kharian Cantt, Pakistan; c Department of Medicine, Kushtia Medical College, Kushtia, Bangladesh; d Department of Medicine, PAF Hospital, Islamabad, Pakistan; e Faculty of Medicine, Mansoura University, Mansoura, Egypt; f Department of Medicine, University College of Medicine and Dentistry, Lahore, Pakistan; g Department of Medicine, Bharati Vidyapeeth Deemed to Be University Medical College, Pune, India; h Department of Radiology, Shifa International Hospital, Islamabad, Pakistan; i Department of Medicine, Federal Medical Center/Baze University, Abuja, Nigeria; j Department of Medicine, People’s University of Medical and Health Sciences for Women, Nawabshah, Pakistan; k Department of Medicine, Liaqat University of Medical and Health Sciences, Jamshoro, Pakistan; l Department of Medicine, Spinghar University, Kabul, Afghanistan; m Faculty of Medicine, Lebanese University, Faculty of Pharmacy, Beirut, Lebanon

**Keywords:** case report, chest wall reconstruction, core needle biopsy, leiomyosarcoma, tumor resection

## Abstract

**Introduction and importance:**

Leiomyosarcomas are extremely rare. Chest wall sarcomas account for less than 20% of all tissue sarcomas, with just 1–4% being leiomyosarcomas. Large leiomyosarcomas often require extensive surgical resection and complex reconstruction. Multidisciplinary management is essential to achieve complete oncologic clearance while restoring chest wall stability and function.

**Case presentation:**

A 43-year-old male presented with a complaint of swelling in the left anterior chest wall found while showering. Examination revealed a 15 × 15 × 10 cm mass in the chest wall. Core needle biopsy and histopathology were consistent with leiomyosarcoma. A multidisciplinary approach involving thoracic and plastic surgery was used to cover the post-resection wound. The tumor was removed, and the resection site wound was covered with latissimus dorsi flaps from the ipsilateral posterolateral aspect of the trunk. The resected specimen weighed 4.5 kg and was sent for histopathological examination to confirm the diagnosis. The patient was discharged with no notable issues post-operatively in the following 30 days; however, he was instructed to attend regular follow-up visits every three months to monitor for disease recurrence.

**Clinical discussion:**

Complete surgical excision remains the primary treatment for localized chest wall leiomyosarcoma. Large defects created after radical resection present significant reconstructive challenges that require multidisciplinary planning. Collaboration between thoracic and reconstructive surgeons facilitates restoration of chest wall integrity, preservation of respiratory function, and acceptable esthetic outcomes.

**Conclusion:**

The case highlights the technical and reconstructive challenges posed by large chest wall sarcomas and underscores the importance of a multidisciplinary approach to achieve oncologic safety while preserving structural integrity, function, and cosmetic aspects.

## Introduction

Soft tissue sarcomas of the chest wall are rare, accounting for less than 20% of all chest wall tumors^[^1^]^. They represent a diverse group of malignant neoplasms with heterogeneous presentations depending on the connective tissue involved. Leiomyosarcoma comprises only 1–4% of chest wall soft tissue sarcomas, making it an especially uncommon entity. Patients typically present with a gradually enlarging, palpable mass and may experience pain due to progressive tumor growth and compression of surrounding structures. Diagnosis is established through a combination of clinical evaluation, cross-sectional imaging, such as CT or MRI, and histopathological confirmation via biopsy.

HIGHLIGHTS

- A middle-aged man presented with a massive leiomyosarcoma of the left anterior chest wall, causing him pain and functional limitations.
- Radiographic imaging showed extensive muscular involvement without bony or intrathoracic extension; the diagnosis was confirmed on biopsy.
- En-bloc tumor resection was performed, which created a large defect; this was reconstructed immediately using an ipsilateral latissimus dorsi flap.
- The collaborative thoracic and plastic surgical approach not only achieved complete tumor removal with negative margins but also provided effective soft-tissue coverage of the thoracic defect.
- Postoperative recovery was smooth, with complete wound healing and no complications, demonstrating successful management of the case.
- The multidisciplinary strategy ensured oncologic safety, chest wall preservation, and improved cosmetic outcomes, demonstrating its value in complex oncologic surgeries.

Surgical excision remains the only definitive treatment and may be supplemented with neoadjuvant chemotherapy or postoperative chemo- or radiotherapy, depending on tumor characteristics. Resection of chest wall tumors generally involves three key phases: achieving wide oncologic margins, reconstructing the chest wall defect, and providing adequate soft tissue coverage, in which plastic surgery plays an essential role^[^2^]^. The limited soft tissue envelope of the chest wall often results in large defects following resection, increasing the risk of exposure of the thoracic organs and chest wall herniation. Additionally, the infiltrative nature of these tumors and their proximity to vital structures make both dissection and reconstruction technically challenging.

Given these complexities, management requires a multidisciplinary approach integrating thoracic surgery, plastic and reconstructive surgery, and radiation oncology. Novel reconstructive techniques, such as the use of a latissimus dorsi (LD) flap, can provide reliable coverage, promote early healing, and enable timely initiation of adjuvant therapy. We highlight a rare case of a large anterior chest wall leiomyosarcoma requiring an extensive, multidisciplinary surgical approach.

This case report is notable for the exceptionally large tumor size (4.5 kg resected specimen), anterior chest wall location, and the need for complex reconstruction using an ipsilateral LD flap following extensive oncologic resection. Massive primary leiomyosarcomas (LMS) of the anterior chest wall are rarely reported in the literature. The case highlights the technical and reconstructive challenges posed by large chest wall sarcomas and underscores the importance of coordinated, multidisciplinary surgical planning to achieve oncologic safety while preserving structural integrity, function, and cosmetic aspects.

This work has been reported according to the SCARE 2025 criteria ^[^3^]^.

## Case presentation

A 43-year-old male school teacher presented to the outpatient department of a tertiary care hospital in Punjab, Pakistan, with a complaint of swelling in the left anterior chest wall. The swelling had been noticed incidentally 2 months earlier during a routine shower. Since then, it had gradually increased in size and become progressively more painful. The pain was physically limiting, interfering with sleep, dressing, and free movement of the left arm in multiple planes.

On general physical examination, the patient appeared well, with only mild discomfort and clinical signs suggestive of anemia. Local examination revealed a large globular swelling on the left anterior chest wall measuring approximately 15 × 15 × 10 cm, involving the left areolar region and the pectoralis major territory (Figs. 1 and 2). The swelling was erythematous, with prominent superficial vessels and crusting of the overlying skin due to stretching. On palpation, it was hard, non-blanchable, slightly warm, non-compressible, non-fluctuant, and immobile. The overlying skin showed areas of crusting but no ulceration. Auscultation revealed no bruit; heart and lung sounds were normal.

**Figure 1.**
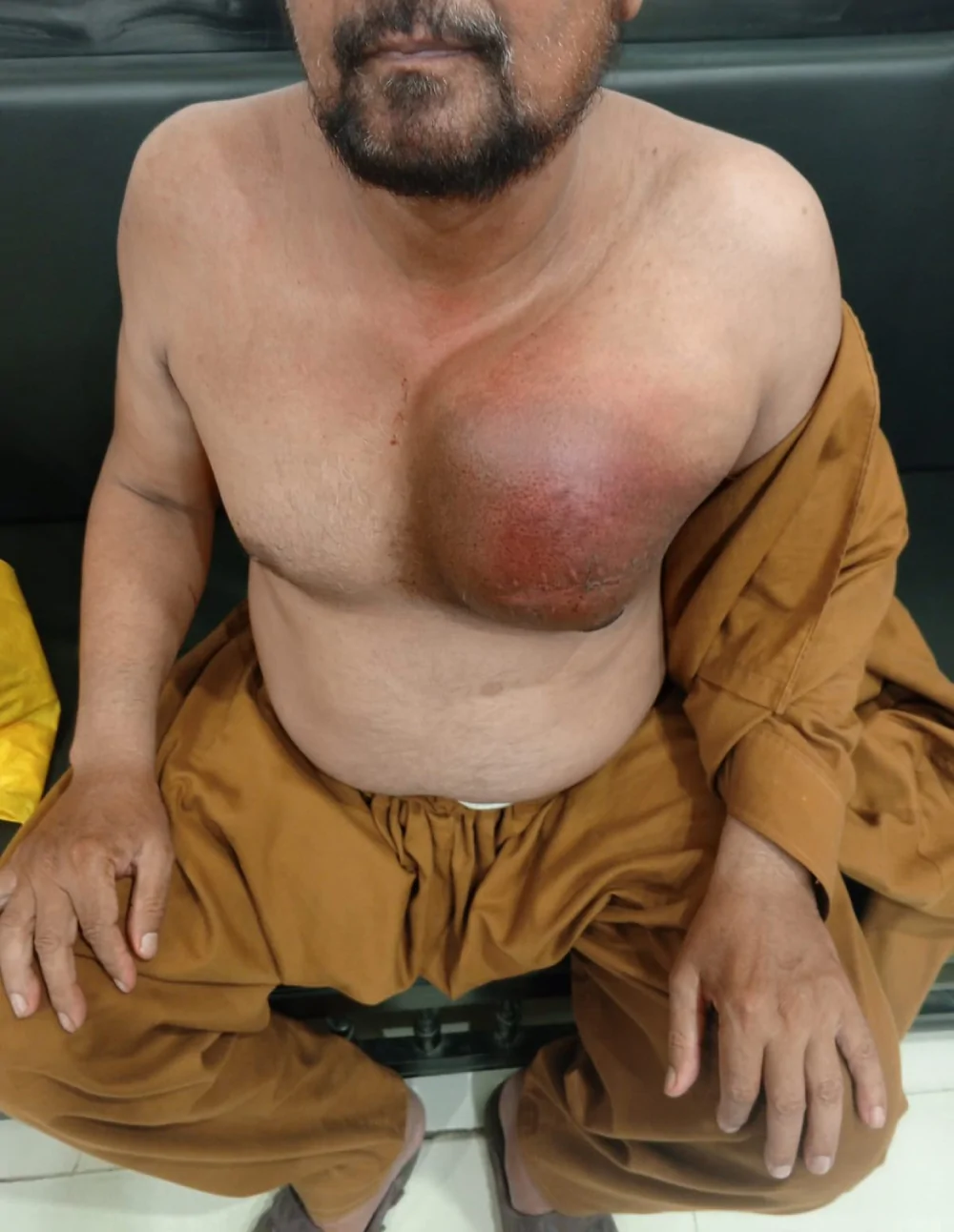
Front view of the chest wall leiomyosarcoma.

**Figure 2.**
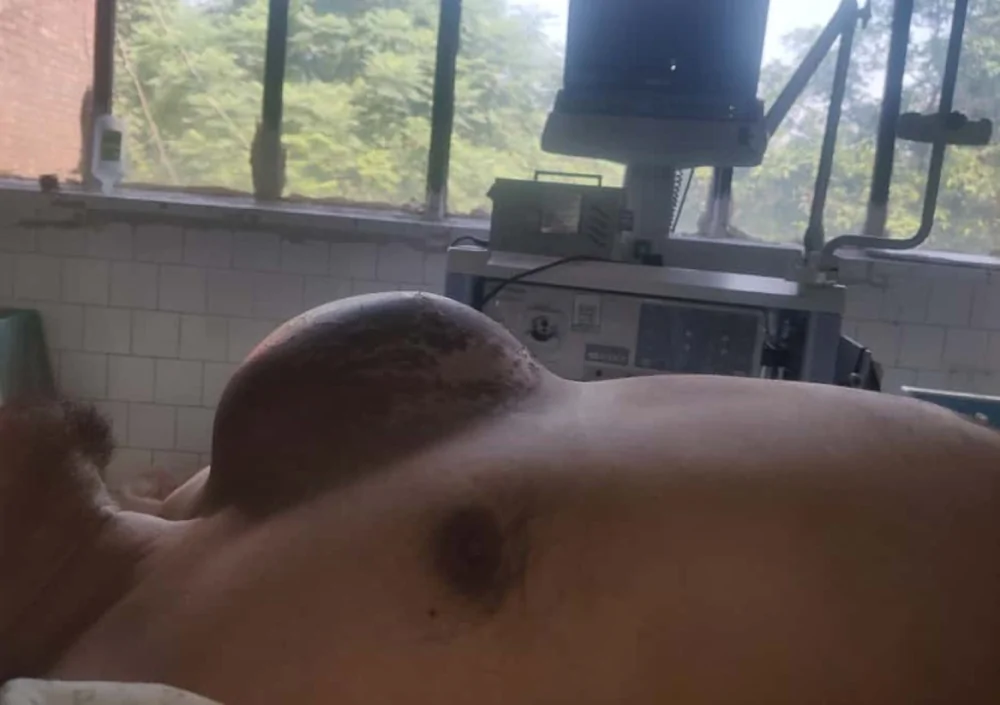
Lateral view of a chest wall leiomyosarcoma.

Chest X-ray and ultrasonography were unremarkable. Contrast-enhanced CT scans of the head (Fig. 3), neck and chest (Fig. 4), and abdomen and pelvis (Fig. 5) were performed to assess the extent of the lesion. Findings demonstrated involvement of the pectoral musculature without bony or intrathoracic extension. Imaging of the chest and abdomen demonstrated a large, localized chest wall mass without radiologic evidence of distant metastasis or regional lymphadenopathy. Based on tumor size (>5 cm), depth, and high-grade histology, the lesion was staged in accordance with the AJCC soft tissue sarcoma staging system as a high-grade, large, deep tumor without distant metastasis. Although lymphatic spread is uncommon in leiomyosarcoma, regional lymph nodes were clinically and radiologically evaluated and showed no evidence of metastatic involvement. High-resolution CT (HRCT) of the chest showed no significant lung pathology (Fig. 6).

**Figure 3.**
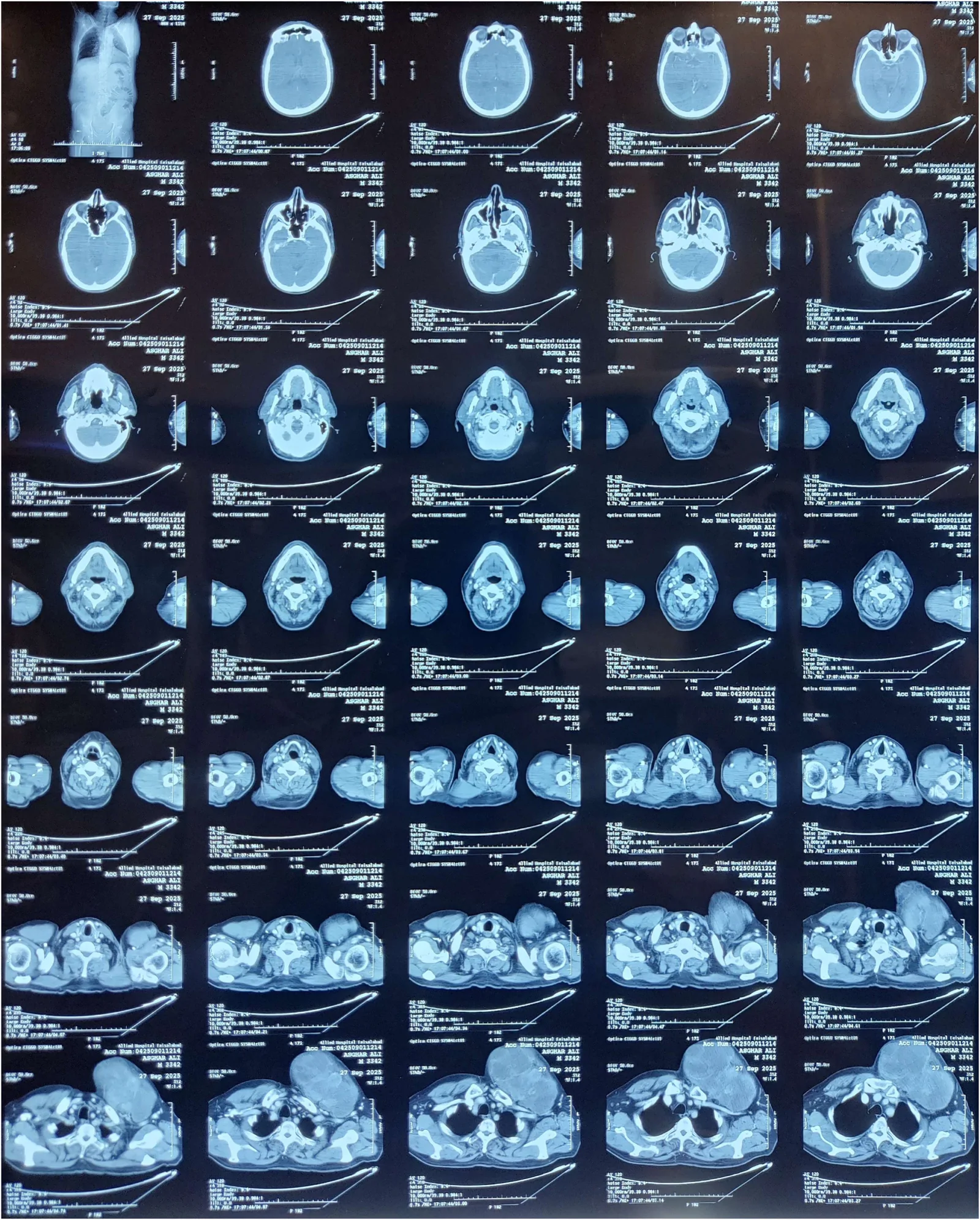
CT scan of the patient's head (findings: normal).

**Figure 4.**
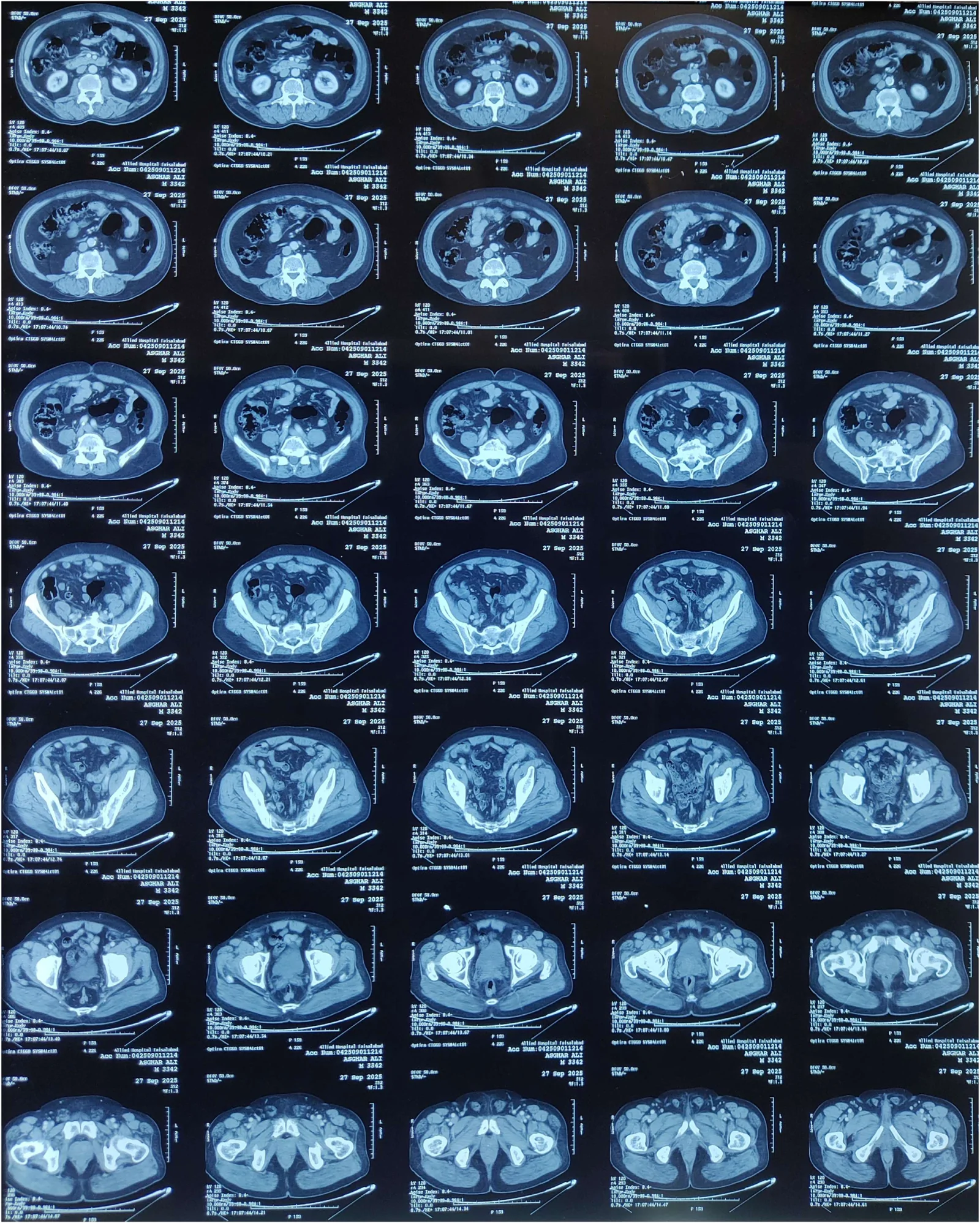
CT scan of the neck and chest of the patient (findings: anterior chest wall mass).

**Figure 5.**
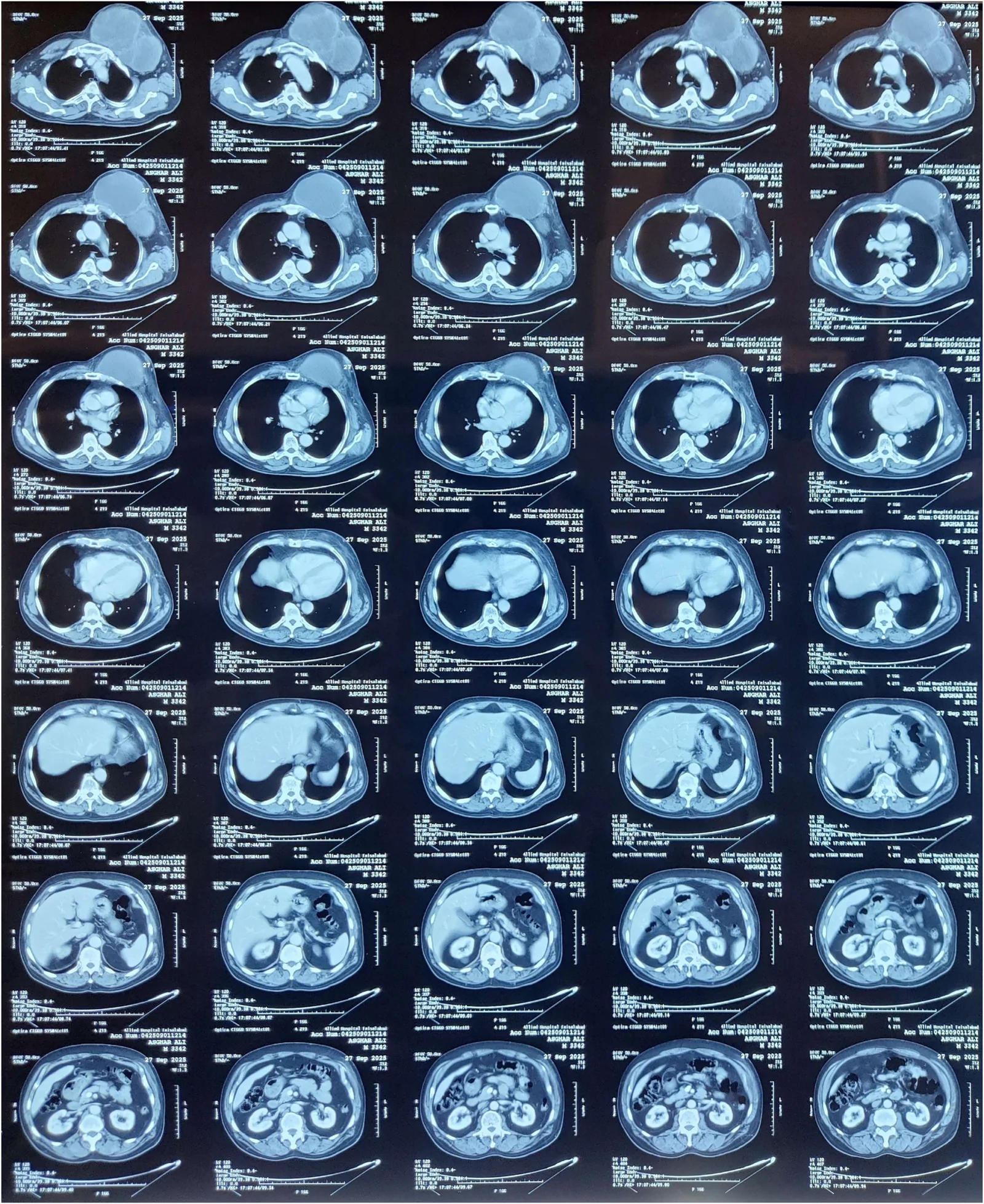
CT scan of the abdomen and pelvis of the patient (findings: normal).

**Figure 6.**
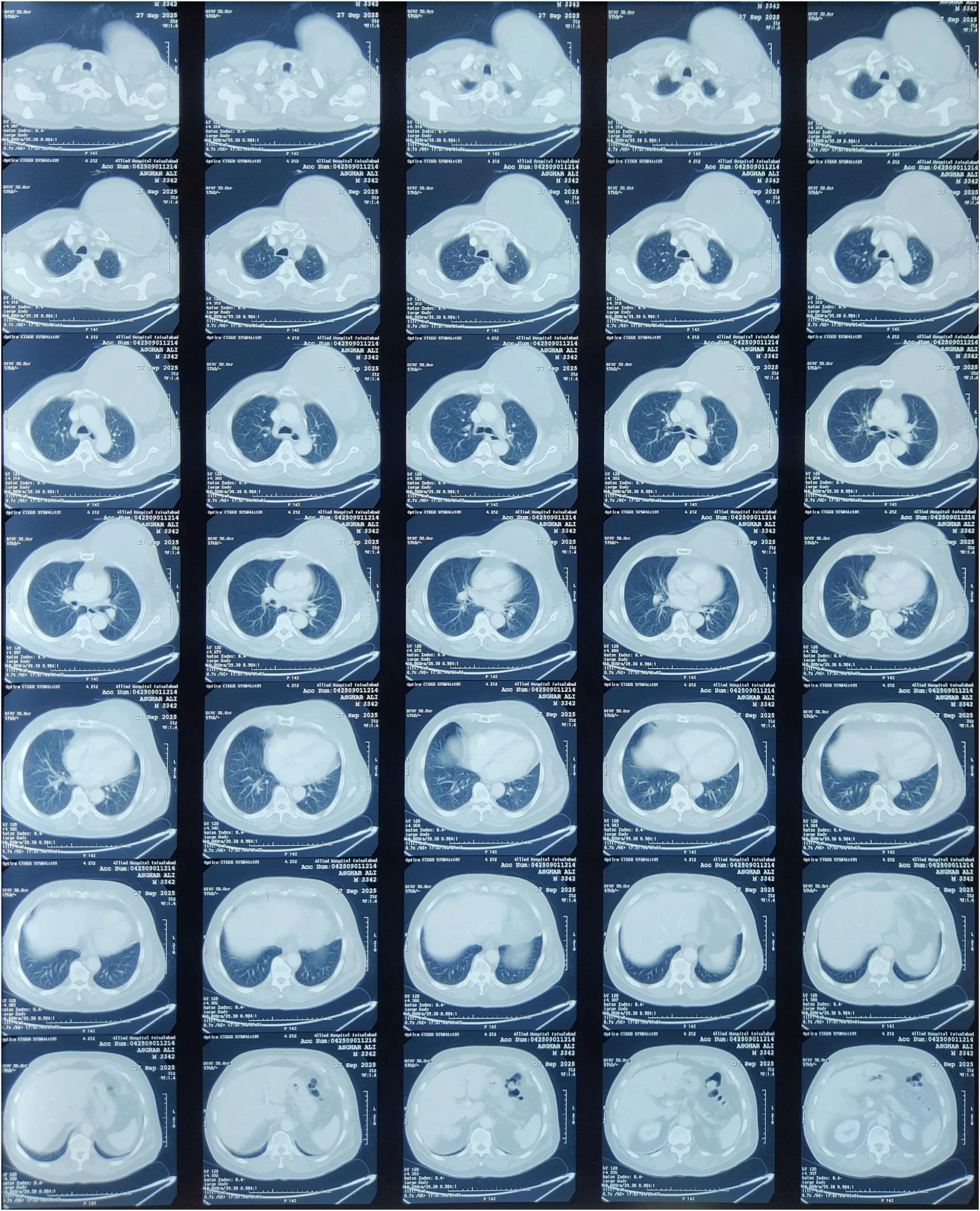
High-resolution CT of the chest (findings: normal).

A core-needle biopsy confirmed leiomyosarcoma. Detailed histopathological evaluation demonstrated a high-grade spindle cell neoplasm arranged in intersecting fascicles, composed of elongated cells with eosinophilic cytoplasm and blunt-ended nuclei. The tumor exhibited marked nuclear pleomorphism, a high mitotic index (>10 mitoses per 10 high-power fields), and focal areas of tumor necrosis. Immunohistochemical staining was strongly positive for smooth muscle actin and desmin, and negative for S-100 and cytokeratin, supporting the diagnosis of high-grade leiomyosarcoma. These findings indicate aggressive biological behavior consistent with the rapid clinical progression observed in this patient.

Given the massive size of the tumor, a multidisciplinary surgical plan was formulated in collaboration with the plastic surgery team. Given the large tumor size and high-grade histology, the case was discussed in a multidisciplinary tumor board setting, including thoracic surgery, plastic surgery, and medical oncology. Neoadjuvant therapy was not administered due to the absence of distant disease and the feasibility of complete surgical resection at presentation. Postoperatively, the patient was referred to medical oncology for evaluation regarding adjuvant therapy. The risks and potential benefits of adjuvant chemotherapy and/or radiotherapy were considered in the context of tumor size, grade, margin status, and current sarcoma treatment recommendations. Based on the biopsy report indicating clear margins and no evidence of metastasis (as explained above in the preoperative stage), the potential risks of adjuvant chemotherapy and/or radiotherapy outweighed the benefits and were not considered; however, the patient was instructed to attend regular 3-month follow-up visits to closely monitor for disease recurrence.

Wide local excision was scheduled, with reconstruction using an ipsilateral LD myocutaneous flap to cover the expected large post-resection defect. The patient was prepared and draped, and the tumor was excised along with the involved overlying skin, ensuring tumor-free margins (Fig. 7a and 7b). Wide local excision with the intent to achieve negative (R0) margins was performed in accordance with established soft tissue sarcoma management principles, which recommend en bloc resection with adequate circumferential margins to reduce local recurrence risk. Given the tumor size and anterior chest wall involvement, resection included involved soft tissue and adjacent structures as necessary to ensure oncologic clearance. Intraoperative assessment and final histopathology confirmed tumor-free margins, aligning with current guideline-based standards for high-grade sarcoma management. The resected specimen weighed 4.5 kg and was sent for histopathology (Fig. 8).

**Figure 7.**
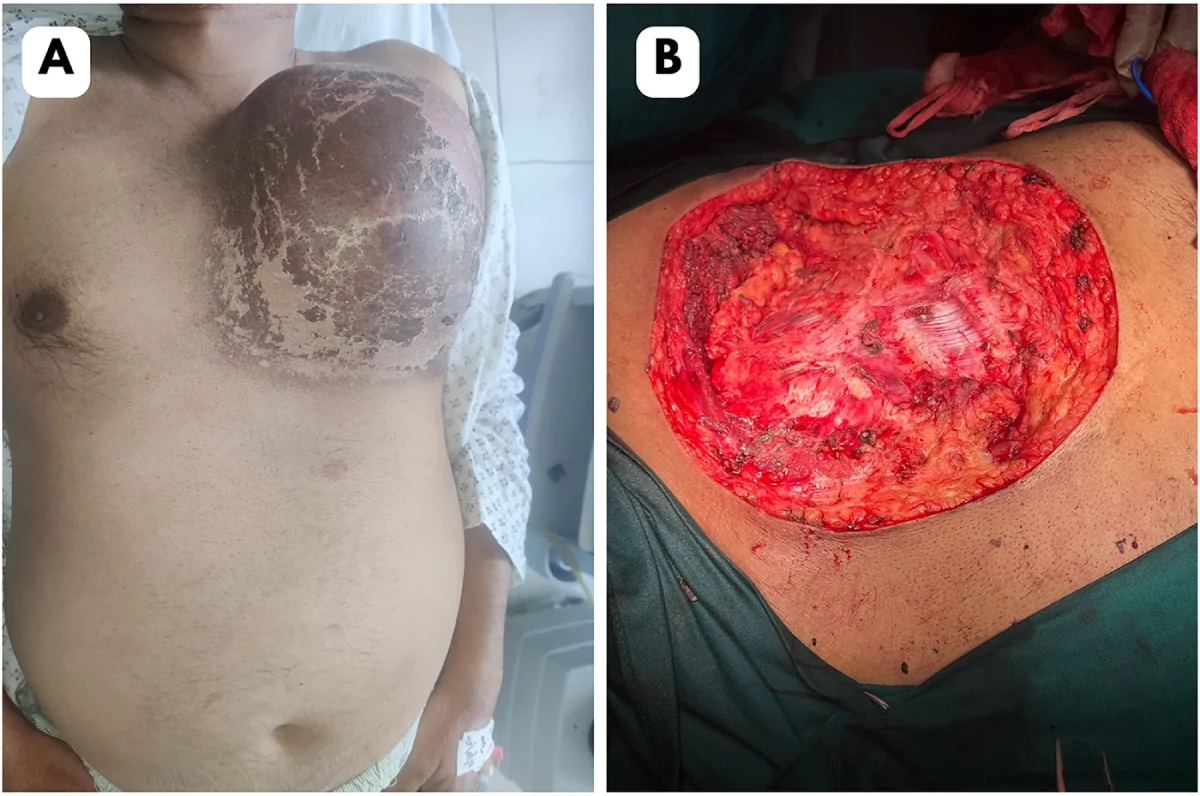
Chest-wall leiomyosarcoma before surgery (a) and after resection (b).

**Figure 8.**
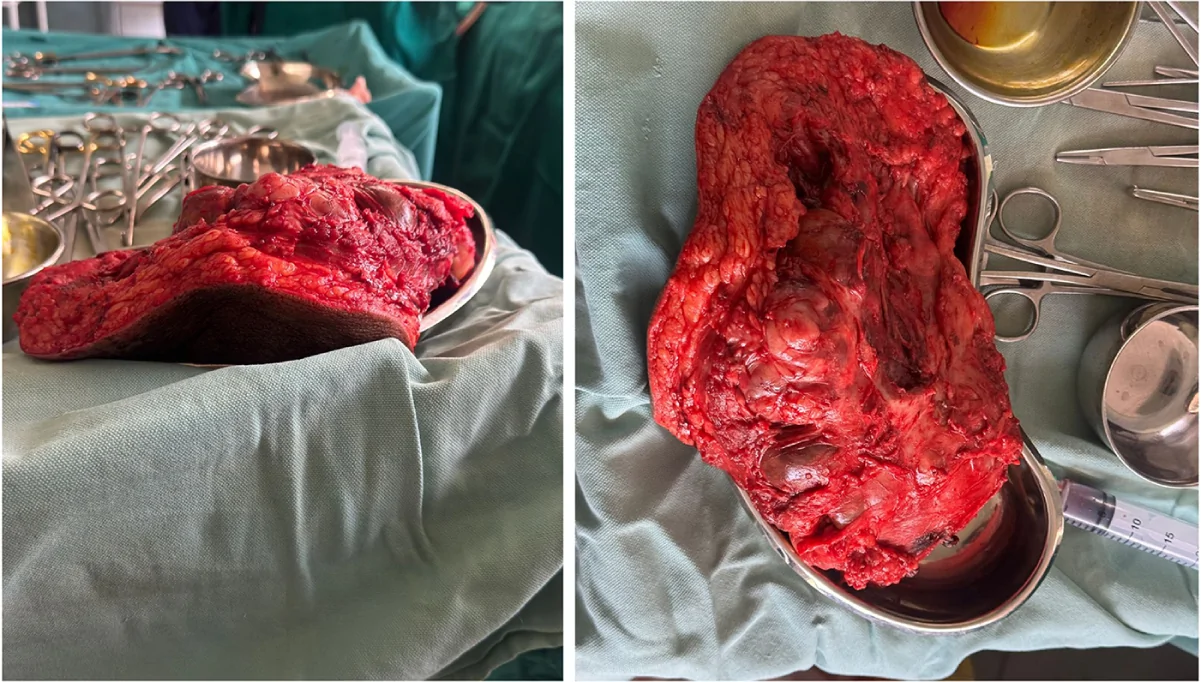
Chest wall leiomyosarcoma specimen removed.

An LD flap was then harvested from the posterolateral aspect of the trunk (Fig. 9). Following hemostasis, the flap was rotated to the recipient site, secured in place; a subcutaneous drain was inserted; and a sterile dressing was applied (Fig. 10). The donor site was closed primarily after achieving hemostasis.

**Figure 9.**
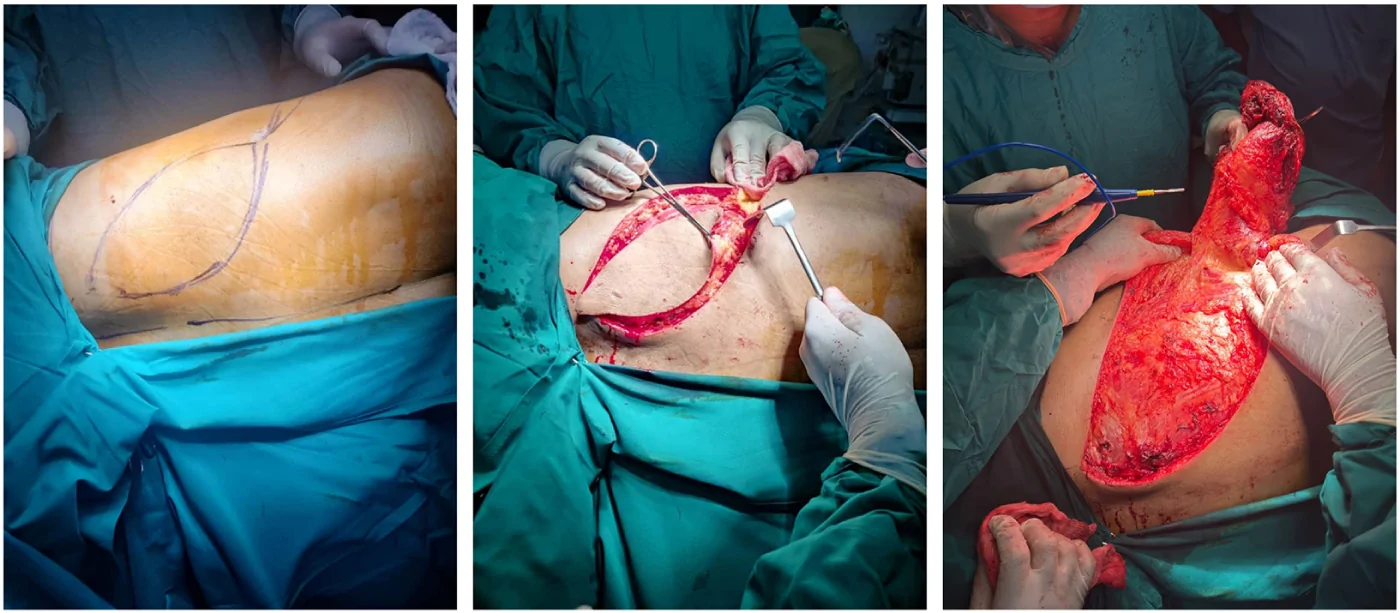
Latissimus dorsi (LD) flap to cover the resection site, shown in sequence: (a), (b), and (c).

**Figure 10.**
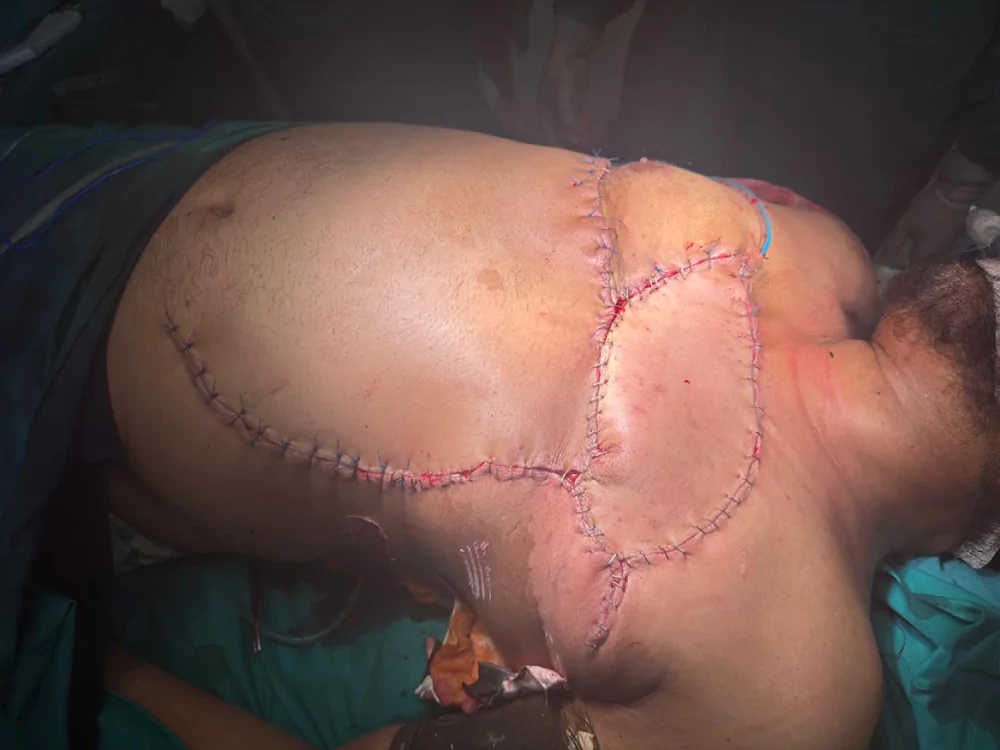
Final image of the resection site after reconstruction with an LD flap.

The patient remained admitted for 4 days for postoperative monitoring. Recovery was unremarkable, with no complications. Histopathology confirmed negative margins, with a minimum clearance of 1 cm at the deepest point. The patient was discharged on postoperative day 5 with instructions regarding wound care, oral analgesics, diet, and activity modification. He was followed up on an outpatient basis at 4 weeks.

A telemedicine review on day 30 post-discharge showed complete recovery with no active complaints. Post-surgical follow-up for wound management was discontinued due to the patient’s stability and healthy wound; however, owing to the high rate of local recurrence and distant metastases, the patient was strictly instructed to attend regular 3-monthly follow-up visits to closely monitor disease recurrence and allow timely intervention in case of recurrence to ensure patient safety.

## Discussion

LMS are malignant neoplasms showing pure smooth muscle differentiation that account for 1–4% of all soft tissue sarcomas and typically appear between 40 and 50 years of age. In the present case, a 43-year-old patient presented with a large leiomyosarcoma of the chest wall, involving only the muscular layer without extension into the bones or adjacent organs. This presentation is consistent with previous reports describing the rarity of primary chest wall leiomyosarcoma, while the exceptionally large tumor size posed unique reconstructive challenges following oncologic resection. Unlike smaller lesions reported in the literature, the extensive post-resection defect in our patient necessitated close collaboration between thoracic and plastic surgery teams to achieve both adequate oncologic clearance and functional chest wall reconstruction. Although soft tissue sarcomas are rare, it is important to consider this diagnosis in patients who seek treatment for tumors of the chest wall. For small superficial tumors, excisional biopsy is a reasonable first diagnostic step. For larger tumors, incisional biopsy allows time for accurate pathologic evaluation. Additionally, preoperative diagnostic tests such as computed tomography or magnetic resonance imaging may be indicated before planning adequate resection^[^4^]^.

In our case, a core needle biopsy was performed to confirm the diagnosis, while CT was used to determine the extent of the disease. This diagnostic approach aligns with current recommendations for evaluating large chest wall soft tissue sarcomas and facilitates appropriate preoperative planning for definitive surgical management. After confirming the diagnosis and assessing the extent of the tumor, the treatment plan depends heavily on evidence from previous studies.

The management of leiomyosarcoma depends on the disease site, tumor size, grade, and patient-dependent factors. Potential options include surgical resection, radiation, and chemotherapy. A multidisciplinary approach at high-volume sarcoma centers is highly recommended, but the primary goals of treatment with curative intent include achieving surgical resection with negative margins, reducing local recurrence, improving functional outcomes, and reducing the risk of distant metastasis^[^5^]^. Even in locally advanced sarcomas or complex cases involving multiple vital structures, surgery seems to provide acceptable perioperative morbidity and mortality, with similar 5-year overall survival to that of more superficial resections^[^6^]^. Our management strategy closely followed these principles, with multidisciplinary surgical planning enabling complete tumor excision while simultaneously addressing the complex reconstructive requirements of the extensive chest wall defect.

Pre- and post-operative radiation therapy can influence surgical margins by biologically sterilizing microscopic extensions of the tumor, potentially reducing the seeding of microscopic sarcomatous cells at the time of surgical resection, and greatly enhancing the ability of the surgeon to achieve negative margins and conserve limb function without compromising disease control^[^7^]^. In a study at The University of Texas M. D. Anderson Hospital, 114 patients treated with preoperative radiation and conservative surgery achieved low primary failure rates, whereas distant metastasis was the main cause of treatment failure in high-grade tumors, predicting poorer survival^[^8^]^.

For the present case of our patient with a leiomyosarcoma measuring 15 × 15 × 10 cm, thoracic surgery was performed for tumor removal, resulting in a large chest wall defect that was reconstructed with an LD flap. In a retrospective study by Sternberg *et al* of 100 consecutive LD flap patients with tissue expander reconstruction (50 immediate and 50 delayed) and a mean follow-up of 2 years, reported complications included the following: 1 patient had partial flap loss, 2 patients required infra-mammary fold revision, and 6 patients had capsular contracture, while donor-site seroma occurred in 34 patients and 6 patients required operative revision^[^9^]^. In another study of breast cancer surgery in Turkey, the LD was used as a musculocutaneous flap in 33 patients who had breast cancer surgery, and 9 patients requested nipple-areola reconstruction, of which 3 patients had major complications (9%), including infection, partial flap ischemia, and liponecrotic pseudocysts, while eight patients required revision, and seroma was the most common problem observed at the donor site^[^10^]^. In a 2-year review of 15 chest wall reconstructions, with defect sizes ranging between 26 × 20 cm and 12 × 15 cm, a hospital stay of 12 days, and follow-up of 6 months to 2 years, there were no major complications except for partial graft loss in only two patients, which healed after a few dressings^[^11^]^. In another case of chest wall reconstruction after chondrosarcoma, a V-Y LD flap was used; after 10 months, there was no evidence of recurrent disease, and the reconstruction appeared acceptable from both functional and cosmetic standpoints^[^12^]^. Our findings are comparable to these reports, as the patient experienced no significant flap-related complications or reconstructive failure despite the large defect requiring reconstruction, and this favorable outcome further supports the reliability of the LD flap for extensive chest wall reconstruction and highlights the benefit of a coordinated thoracic and plastic surgical team approach in managing complex oncologic defects.

Complications of chest wall resection and reconstruction range from mild to life-threatening and can be broadly categorized into surgical-site complications, which largely pertain to the surgical technique and prosthetic materials (e.g., infections, hematomas, seromas, and dehiscence), respiratory complications, and other systemic complications that may arise as a result of a major procedure. Several of these complications can have a consequential impact on the patient’s recovery, functional capacity, and quality of life. Therefore, prevention, prompt identification, and appropriate management of such complications are crucial for successful outcomes^[^13^]^. The absence of major postoperative complications in our patient further demonstrates that meticulous multidisciplinary planning and appropriate reconstructive techniques can facilitate favorable early postoperative outcomes, even after resection of large chest wall LMS.

The growing integration of large language models (LLMs) such as ChatGPT in medical research has expanded their use in manuscript drafting, literature synthesis, and clinical decision support, while raising concerns regarding accuracy, transparency, ethical considerations, and the need for human oversight. In rare oncologic conditions such as massive chest wall leiomyosarcoma, LLMs may enhance scientific communication but cannot substitute for multidisciplinary evaluation, individualized surgical planning, or expert histopathological assessment. Clinical decision-making must therefore remain grounded in validated evidence, established guidelines, and specialist expertise^[^14^]^.

While working on this manuscript, LLM tools were used for language and grammar purposes only. All intellectual content and information are based solely on the authors’ expertise, knowledge, and evidence-based medical literature.

## Limitations

We acknowledge that the current follow-up duration for post-surgical wound management is limited to the early postoperative period; however, the patient was strictly instructed to attend regular 3-monthly follow-up visits to closely monitor disease recurrence and intervene promptly if necessary, ensuring the patient’s safety.

Given the high risk of local recurrence and distant metastasis associated with high-grade leiomyosarcoma, an ideal surveillance strategy would include clinical examination and cross-sectional imaging of the chest every 3–6 months for the first 2–3 years, followed by annual surveillance thereafter, in accordance with soft tissue sarcoma follow-up recommendations. Long-term monitoring is essential for early detection of recurrence or metastatic progression.

However, in lower- and middle-income countries, where electronic health record systems and remote follow-ups are in the early stages of development, prolonged follow-up is difficult. Public attitudes toward routine checkups and prolonged follow-up (when there is no visible medical issue) are poor, which further exacerbates the problem. Therefore, while using this case report in clinical practice, it is important to acknowledge that prolonged follow-up is necessary in such cases owing to the high risk of local recurrence and distant metastasis.

Additionally, as a single case, it cannot be generalized to a broader patient population with chest wall LMS. Moreover, due to the rarity of these tumors, this multidisciplinary approach cannot be compared with other strategies.

When viewed in context, our experience reinforces the importance of a coordinated thoracic and plastic surgery approach for managing extensive chest wall defects following leiomyosarcoma resection.

## Conclusion

This case highlights the complexity of managing massive chest wall leiomyosarcoma, demonstrating that a multidisciplinary approach, combining thoracic and plastic surgery, is highly recommended for better outcomes. Early diagnosis, complete tumor resection, and meticulous reconstruction not only restore structural integrity but also improve patients’ quality of life. This case reinforces the importance of coordinated surgical planning and individualized strategies in addressing large, challenging thoracic tumors.

## Data Availability

Not applicable.
